# An Evaluation of the Performance and Acceptability of Three LED Fluorescent Microscopes in Zambia: Lessons Learnt for Scale-Up

**DOI:** 10.1371/journal.pone.0027125

**Published:** 2011-11-04

**Authors:** Eleanor R. Turnbull, Kaunda Kaunda, Jennifer B. Harris, Nathan Kapata, Mweemba W. Muvwimi, Annika Kruuner, German Henostroza, Stewart E. Reid

**Affiliations:** 1 Centre for Infectious Disease Research in Zambia, Lusaka, Zambia; 2 Schools of Medicine and Public Health, University of Alabama at Birmingham, Birmingham, Alabama, United States of America; 3 National TB/Leprosy Program, Zambian Ministry of Health, Lusaka, Zambia; 4 Chest Diseases Laboratory/National Tuberculosis Reference Laboratory, Zambian Ministry of Health, Lusaka, Zambia; San Francisco General Hospital, University of California San Francisco, United States of America

## Abstract

The World Health Organization recommends the roll-out of light-emitting diode (LED) fluorescent microscopes (FM) as an alternative to light microscopes in resource-limited settings. We evaluated the acceptability and performance of three LED FMs after a short orientation among laboratory technicians from government health centers in Zambia. Sixteen technicians with varied light microscopy experience were oriented to FMs and divided into groups; each group read a different set of 40 slides on each LED FM (Primo Star iLED™, Lumin™, FluoLED™) and on a reference mercury-vapor FM (Olympus BX41TF). Slide reading times were recorded. An experienced FM technician examined each slide on the Olympus BX41TF. Sensitivity and specificity compared to TB culture were calculated. Misclassification compared to the experienced technician and inter-rater reliability between trainees was assessed. Trainees rated microscopes on technical aspects. Primo Star iLED™, FluoLED™ and Olympus BX41TF had comparable sensitivities (67%, 65% and 65% respectively), with the Lumin™ significantly worse (56%; p<0.05). Specificity was low for trainees on all microscopes (75.9%) compared to the experienced technician on Olympus BX41TF (100%). Primo Star iLED™ had significantly less misclassification (21.1% p<0.05) than FluoLED™ (26.5%) and Lumin™ (26.8%) and significantly higher inter-rater reliability (0.611; p<0.05), compared to FluoLED™ (0.523) and Lumin™ (0.492). Slide reading times for LED FMs were slower than the reference, but not significantly different from each other. Primo Star iLED™ rated highest in acceptability measures, followed by FluoLED™ then Lumin™. Primo Star iLED™ was consistently better than FluoLED™ and Lumin™, and performed comparably to the Olympus BX41TF in all analyses, except reading times. The Lumin™ compared least favorably and was thought unacceptable for use. Specificity and inter-rater reliability were low for all microscopes suggesting that a brief orientation was insufficient in this setting. These results provide important data for resource-limited settings to consider as they scale-up LED FMs.

## Introduction

Zambia, a country of about 13 million people [1], has an annual tuberculosis (TB) incidence rate of 433/100,000) [2]. Seventy percent of TB patients are co-infected with HIV [3] and TB is a leading cause of death in co-infected patients [4]. Prompt and accurate diagnosis of TB is particularly critical in HIV infected patients to reduce the associated morbidity and mortality; determine the most appropriate treatment; prevent the development of immune reconstitution inflammatory syndrome (IRIS), and reduce transmission in health care facilities and in the community. However, TB diagnosis is challenging, especially in HIV infected patients, and laboratory diagnostics have been identified as the weakest part of most TB programs.

Sputum smear examination using light microscopy and Ziehl-Neelsen (Z-N) staining is Zambia's primary TB diagnostic method. Although highly specific, this method has low sensitivity, particularly in patients with low concentrations of mycobacteria [5] which is common in HIV-positive individuals [6]. Thus the usefulness of Z-N microscopy is limited in high HIV prevalence settings like Zambia, where the World Health Organization (WHO) estimates that only 58% of the smear positive cases are detected [7]. Fluorescent microscopy is known to perform significantly better than Z-N microscopy, both in reading time and sensitivity [5], [8], [9]. Furthermore the increased sensitivity of FM over light microscopy is strongest in paucibacillary cases; important for diagnosing TB in HIV infected patients [5], [10]. However mercury vapor fluorescent microscopes, such as the Olympus BX41TF, may not be feasible for use or affordable in low resource countries such as Zambia [11].

The WHO has recently recommended the roll out of low cost light emitting diode (LED) fluorescent microscopes (FM) in resource-limited settings as an alternative to the current light microscopes and mercury vapor FMs [12]. Studies conducted in reference laboratory settings, showed LED FMs to have an average of 10% greater sensitivity than light microscopy and similar specificity [13], [14], [15]. LED fluorescent microscopes are inexpensive, use cheap and affordable bulbs with life spans greater than 10,000 hours; can run on batteries and do not require a dark room [16], [17], [18], [19], [20], [21], [22]. In addition, studies have found that LED FMs have 2–4 times faster examination time per slide [14], [23], [24]. This is critical for countries with health care worker shortages; Zambia is functioning with only 27% of its needed laboratory staff [25].

To date the majority of LED FM literature focuses on validation studies conducted in reference laboratories using laboratory technicians who are well-trained and experienced in fluorescent microscopy [14], [19], [20], [26]. As a next step, WHO recommends country-specific adaptation and validation of LED FMs. In this study, registered technicians from government health centers in Lusaka, Zambia, with a two year diploma in medical laboratory technology or biomedical sciences, and experience in Z-N microscopy were given a short orientation to fluorescent microscopy. They were then asked to (a) examine slides on three different LED FMs and on a reference mercury-vapor FM and (b) complete a subjective evaluation of each microscope in order to assess the initial acceptability and suitability of these LED FMs for use in Zambian health centers. This study evaluated which LED FM could produce acceptable end results in technicians with very little prior FM exposure and can be used to guide recommendations on the roll out of LED FMs in Zambia.

## Methods

### Ethics Statement

This protocol was approved by the institutional review boards of the University of Zambia, Protocol # 009-11-08 (Lusaka, Zambia), the University of Alabama at Birmingham, Protocol # N090210004 (Birmingham, Alabama, USA) and the Zambian Ministry of Health. The requirement for obtaining consent from patients was specifically waived by the appropriate institutional review boards because only anonymous sputum specimens, collected as part of routine clinical care, were used and no identifying characteristics of patients were recorded. All technicians reviewed an information sheet describing the study, which had been previously approved by the institutional review boards, before verbally consenting to participate.

### Specimen collection, preparation and processing

Sputum specimens were obtained from TB suspects at Kalingalinga District Health Center in Lusaka between January–July 2010. Specimens were anonymous and labeled only with a patient and specimen number. Patient HIV status was recorded after reviewing documentation of enrollment into HIV care or an HIV test within the last 6 months. As per National guidelines, three specimens were provided from each patient and a Z-N stained slide was made and examined for each specimen; results were reported back to clinicians so that patient care was unaffected. A leftover of the specimen was used for TB culture and storage. All specimens were cultured on both solid media (Lowenstein-Jensen method [27]) and liquid media using the automated BD Bactec MGIT 960 system [28]. All acid-fast bacilli (AFB) positive cultures that were also cord factor positive by ZN staining were confirmed as *M. tuberculosis* complex with MPT 64 antigen test (MGIT TBc Identification test, Becton Dickinson) while those without cording were identified using the GenoType® Mycobacterium CM assay (Hain Lifesciences). 0.5 to 1 ml of raw specimen was stored at −20 degrees Celsius in a specimen repository at the Centre for Infectious Disease Research in Zambia (CIDRZ) reference laboratory in Lusaka.

In January 2011, eighteen sputum specimens were selected from the sputum repository and used to make 160 slides with Auramine-O stain. Twelve specimens were culture positive and used to make 111 slides; six specimens were culture negative and used to make 49 slides. All slides were made from direct sputum. Each batch of forty slides was made in one day and examined within 32 hours of staining. The slides were made with varying quality smears, half of good quality (n = 80) and half of poor quality (too thin (n = 40) or too thick (n = 40)) by an experienced reference laboratory technician to replicate the varying quality of slides encountered in usual health center settings. Slides were bar-coded so that results from different FMs could not be compared during reading. The barcode number linked slides to MGIT culture result, HIV status and slide quality.

### Microscope Evaluation

Three LED FM systems were chosen for this evaluation based on recommendations from experts in the field and availability at the time the study was designed. The evaluated microscopes were: (a) Primo Star iLED™ (Carl Zeiss Microimaging, Oberkochen, Germany), a stand-alone microscope with reflected light source [29]; (b) Lumin™ (LW Scientific, Lawrenceville, GA, USA), an LED objective adaptor using reflected light source which was mounted on a Olympus CX41 light microscope [30]; and (c) FluoLED™ - AFTERH [Amplified Fluorescence (by) Transmitted Excitation (of) Radiation] LED fluorescence add-on kit (Fraen SRL, Settimo, Italy), using transmitted light [31] mounted on a Olympus CX41 light microscope. An Olympus BX41TF mercury-vapor FM was used as the reference microscope.

Sixteen technicians from ten government health center laboratories in Lusaka, Zambia were chosen by the Ministry of Health. Fifteen out of sixteen technicians had no prior training or experience with LED fluorescent microscopy. The remaining technician had had one week of training and two months of experience. They had between 9 months and 22 years of experience using light microscopes and examined an average of 83 Z-N slides per week at their respective laboratories. After verbally consenting to participate, the technicians underwent a short orientation on fluorescence microscopy, with three hours of didactic learning and two hours hands-on practice focusing and reading slides on each microscope. Didactic topics covered were: smear preparation and FM staining methods; reading and reporting of fluorescent smears; use and maintenance of LED microscopes/attachments; and fluorescence microscopy quality assurance. FM staining was not practiced. Due to the limited number of microscopes, trainee technicians were later divided into groups of four to conduct the evaluation. Over two consecutive days, each group examined 40 slides on each of the four different microscopes, totaling 160 slide readings per trainee. To avoid re-staining slides, each group of trainees read a different set of 40 slides.

A senior technician with 3.5 years of FM experience, employed at the reference laboratory, re-examined all of the slides on the Olympus BX41TF to provide a ‘reference standard’ against which the district technicians' readings on the LED FMs were evaluated.

Fluorescent smears were examined at 400× magnification with all four microscopes. Grading of smears was according to WHO/IUATLD guidelines [27] for fluorescent microscopes and a grading chart was available for technician review during slide reading. Readings moved across one length of the smear and technicians were instructed to complete examination of the following before reporting a result: 40 fields for a smear negative, scanty or 1+ result; 20 fields for a 2+ result; and 8 fields for a 3+ result. Technicians used a digital timer to record time-to-determination of acid-fast bacilli result for each slide starting from the moment the slide was focused under the microscope. All readings with LED FMs were conducted in a room with natural light, whilst readings on the Olympus BX41TF were performed in a darkened room.

Sets of ten slides were stored in slide boxes and numbered from 1–10. Technicians were instructed to read one ten-slide panel on each microscope and record results on the study-specific form beside the appropriate number (1–10). Separate forms were used for each panel reading and documented the technician ID number, microscope and panel number. After finishing the panel, the form was given to the study coordinator who un-coded the slides and linked the reader's result to the actual slide ID. After a panel of slides had been read by all four technicians on the same microscope it was moved to the next microscope and the order of the slides was randomly changed before the technicians started the next round of reading. This process was repeated three times so that each trainee did 160 readings (four panels on each of the four microscopes). Technicians were blinded to the previous slide results and were not aware that they were re-reading the same slides on a different microscope.

After completing all slide readings the trainee technicians completed a subjective questionnaire assessing initial experience and impressions and rating each microscope with a scale of 1–5 (1 = very bad; 5 = very good) in the following six categories: adaptability of viewing height; focus mechanism; contrast and colour impression; homogeneity of fluorescence illumination; resolution of focus; and depth of focus. In addition, they ranked the microscopes in order of preference for their use in the government health centers. Questionnaire terminology was reviewed as a group prior to completion to ensure full comprehension.

All data was entered into a Microsoft Access database by a data technician.

### Outcomes of interest

Outcomes of interest included sensitivity and specificity, the proportion of slides misclassified, inter-rater reliability between technicians, the mean time required to read a slide, and the technician's subjective rating of each microscope. Sensitivity and specificity were calculated using TB culture results as the gold standard. To measure misclassification, slide reading results from both the trainee technicians and the experienced reference laboratory technician were categorized as ‘positive’ (including scanty, 1+, 2+ and 3+ results) or ‘negative.’ The experienced laboratory technician's result was considered the reference standard and trainee results that differed were considered ‘misclassified.’ Inter-rater reliability was evaluated among the trainee technicians using a weighted kappa which takes into account all five possible reading results as an ordinal scale (negative, scanty, 1+, 2+, 3+) and assigns greater weight to results that are further apart from each other on the scale. The time required by trainee technicians to examine each slide was self-recorded in seconds. Subjective rating of the microscopes was measured with a five point ordinal scale (1 = very bad; 5 = very good). Mean time to examine a slide and subjective rating of the microscopes were assessed for the Olympus BX41TF but were not included in the data tables as this study was evaluating which LED FM was most appropriate for use in district laboratories in Zambia.

### Statistical Analyses

#### Sensitivity and specificity

The sensitivity and specificity for the reference technician on the Olympus BX41TF compared to culture results (as the gold standard) were calculated from a frequency table. For the trainee technicians, sensitivity of their slide reading results compared to culture was calculated for each technician on each microscope from the 40 slide readings they did per microscope. These sensitivities were then used as the dependent variable in a linear mixed model using the SAS PROC MIXED procedure with default restricted maximum likelihood estimation (REML). The only fixed effect was the microscope used (Primo Star iLED™, FluoLED™, Lumin™, Olympus BX41TF). The trainee technician was included as a random effect to account for within-person clustering. Mean sensitivities for the microscopes were estimated from the least squares means of the fixed effect. An omnibus F test was conducted to see if there was a significant difference between any of the microscopes. When the omnibus test was significant, pair-wise comparisons between microscopes were conducted. All tests were two-sided with α = 0.05. These procedures were repeated with specificity as the dependent variable.

#### Misclassification

Percent misclassification of the trainee technicians' readings as compared to the reference technician's readings was calculated for each technician on each microscope. A linear mixed model was then developed and evaluated as described above.

#### Inter-rater reliability

A weighted kappa was calculated for each pairing of technicians that read the same group of 40 slides. For example, the first 40 slides were read by technicians 1–4; so a weighted kappa was calculated between technicians 1&2, 1&3, 1&4, 2&3, 2&4, and 3&4 for each of the four microscopes. After doing this for all four groups, there were 24 weighted kappas per microscope, each associated with a pair of technicians. A linear mixed model was developed with weighted kappa score as the independent variable and microscope as the fixed dependent variable. The technician pair (e.g. technicians 1&2) was included as a random effect to account for clustering within each pair. An omnibus test and pair-wise comparisons were conducted as described above.

#### Examination time

A linear mixed model with reading time as the dependent variable was developed with each reading as one observation. The fixed effect was the microscope used (Primo Star iLED™, FluoLED™, Lumin™, Olympus BX41TF). Random effects included the trainee technician and their group to account for within-person and within-group clustering. Slide number was included as a repeated effect to account for multiple readings of each slide by different trainees. An omnibus F-test and pair-wise comparisons were conducted following the same procedures as in the misclassification model.

#### Sub-group analyses

For misclassification, inter-rater reliability and examination time, sub-group analyses were conducted for the following groups: (1) good quality slides; (2) poor quality slides; (3) slides from HIV positive patients; and (4) slides from HIV negative patients. For the outcome of examination time, additional sub-group analyses were conducted on (1) negative slides; (2) low positive (scanty, 1+) slides; and (3) high positive (2+, 3+) slides.

#### Sensitivity analyses

Per study protocol, all trainee technicians were included in the primary results. However, three trainee technicians performed well below the standard of the other technicians suggesting that they either (a) did not understand information provided in orientation or (b) did not follow study protocol. In case the latter is true, sensitivity analyses were conducted using the same models described above but excluding results from these three technicians.

#### Subjective rating of microscopes

A mixed linear model was developed for each question and for the overall mean rating of the microscopes. The score allocated to the question (1 = very bad; 5 = very good) was the independent variable with microscope as the fixed effect. Technician number was included as a random effect to account for within-person clustering in the technicians' rating of each microscope. An omnibus F test and pairwise comparisons were conducted as described above.

All analyses were performed using SAS Software, version 9.2 (Cary, North Carolina, USA).

## Results

The reference laboratory technician graded 153 slides on the Olympus BX41TF microscope as follows: 80 negative, 16 scanty, 17-1+, 18-2+ and 22-3+. An additional 7 slides were broken during the study and thus not examined by the reference laboratory technician. Using the Olympus BX41TF microscope, slide examination by the reference technician had a sensitivity of 61.9% and specificity of 100%. Sensitivity and specificity for smear microscopy by the trainee technicians are shown in Table 1. The Lumin™ had a significantly lower sensitivity (55.8%) than the FluoLED™ (65.1%; p<0.05), the Primo Star iLED™ (67.0%; p<0.05) and the Olympus BX41TF (65.2%; p<0.05). There were no significant differences in specificity between prototypes; however there was a large difference in the specificity of the Olympus BX41TF for trainee technicians (75.4%) compared to the experienced technician (100%). In a sensitivity analysis that removed the three technicians who had performed poorly compared to the rest of the group, sensitivity decreased and specificity increased on all prototypes. This is because the three technicians heavily over-reported positive results.

**Table 1:** Sensitivity and specificity of prototypes with examination by 16 trainee technicians when compared to TB culture.

All Slides (N = 160)	Primo Star iLED™	FluoLED™	Lumin™	Olympus BX41TF
Sensitivity	67.0%^L^	65.1%^L^	55.8%	65.2%^L^
Specificity	74.4%	74.0%	79.9%	75.4%
Sensitivity excluding 3 technicians#	59.8%^L^	57.6%^L^	49.1%	57.4%^L^
Specificity excluding 3 technicians#	89.1%	87.8%	91.9%	88.9%

L Performed significantly better (p<0.05) than Lumin™.

# A sensitivity analysis was conducted excluding three readers who had misclassification rates >40%.

In this evaluation the Primo Star iLED™ had significantly less misclassification (21.1%) than both the FluoLED™ (26.5%; p<0.05) and the Lumin™ (26.8%; p<0.05) in all slides (Table 2). In sub-group analyses, the Primo Star iLED™ also had the lowest misclassification rate in HIV positive slides, and performed significantly better than both Lumin™ and FluoLED™ (p<0.05). The Primo Star iLED™ compared similarly in misclassification rates to the Olympus BX41TF (21.1% vs. 20.8%), whilst the FluoLED™ and the Lumin™ both had inferior performance compared to the Olympus BX41TF.

**Table 2:** Percentage of slides misclassified (positive or negative) by sixteen trainee technicians when compared to an experienced reference laboratory technician.

Type of slide (N)	Primo Star iLED™	FluoLED™	Lumin™	Olympus BX41TF
All slides (153*)	21.1%^F^^,^ L	26.5%	26.8%	20.8%^F^^,^ L
Good quality (77)	18.6%^F^	28.9%	24.7%	23.1%
Poor quality (76)	23.5%	24.1%	28.9%	18.5%^L^
HIV positive (96)	25.4%^F^^,^ L	33.0%	32.2%	26.3%^F^
HIV negative (57)	13.8%	15.6%	17.8%	11.6%
All Slides (153) excluding 3 technicians#	14.7%^F^ ^L^	20.6%	23.2%	14.3%^F^ ^L^

F Performed significantly better (p<0.05) than FluoLED™;

L Performed significantly better (p<0.05) than Lumin™.

*7 slides were broken and not read by the experienced reference laboratory technician, and are thus excluded from this analysis.

# A sensitivity analysis was conducted excluding three readers who had misclassification rates >40%.

Individual technician misclassification rates (data not shown) indicated that three readers were particularly poor with misclassification rates of 43%, 48% and 54%. A sub-analysis of misclassification by microscope without the three poor readers reduced all rates of misclassification but did not affect overall trends (Table 2).

When compared to the reference technician readings, trainee technicians were more likely to report false positive as opposed to false negative results on all microscopes. The proportion of misclassified results that were false positive was 73.2% on the Primo Star iLED™, 66.7% on the FluoLED™, 51.2% on the Lumin™ and 70.3% on the Olympus BX41TF. However, after removal of the three readers with abnormally high misclassification, this pattern disappeared. The percentage of misclassified slides that were false positive decreased to 51.4% on the Primo Star iLED™, 49.0% on the FluoLED™, 33.3% on the Lumin™ and 48.6% on the Olympus BX41TF.

To determine misclassification in Table 2, readings were assessed dichotomously (positive or negative). However, examination of results on the full grading scale (data not shown) found that the majority of misclassified results were discrepancies between negative and scanty readings. The proportion of misclassified readings that were negative/scanty discrepancies was 53.6% on the Primo Star iLED™, 64.8% on the FluoLED™, 68.3% on the Lumin™ and 68.3% on the Olympus BX41TF. After removal of the three readers with abnormally high misclassification, the proportion of misclassified slides that were negative/scanty discrepancies increased for all prototypes except the Lumin™. The revised proportions were 85.7% on the Primo Star iLED™, 68.4% on the FluoLED™, 65.8% on the Lumin™ and 68.6% on the Olympus BX41TF

The Primo Star iLED™ showed a significantly higher overall inter-rater reliability (0.611; Table 3) compared to the other LED FMs: FluoLED™ (0.523; p<0.05) and Lumin™ (0.492; p<0.05). In sub-group analyses, similar differences were found among poor quality and HIV positive slides but there were no significant differences between LED FMs in good quality slides. Among HIV negative slides, the Primo Star iLED™ performed significantly better than the Lumin™ but not the FluoLED™. Inter-rater reliability on both the FluoLED™ and Primo Star iLED™ was not significantly different from the Olympus BX41TF. A further sensitivity analysis was conducted removing all readings from 3 technicians who had consistently high rates of misclassification. The weighted kappa scores for all slides were consistently higher in this sub-analysis, but trends for the microscopes remained the same, with the Primo Star iLED™ remaining superior to the FluoLED™ and Lumin™ (0.705 vs. 0.627 and 0.546; p<0.05).

**Table 3:** Mean inter-rater reliability (weighted kappa statistic) between pairings of trainee technicians that read the same group of forty slides.

Type of slides (N)	Primo Star iLED™	FluoLED™	Lumin™	Olympus BX41TF
All slides (160)	0.611^F^^,^ L	0.523	0.492	0.577^L^
Good quality (80)	0.650	0.569	0.580	0.565
Poor quality (80)	0.557^F^^,^ L	0.459^L^	0.339	0.581^F^^,^ L
HIV positive (100)	0.561^F^^,^ L	0.421	0.398	0.489^L^
HIV negative (60)	0.630^L^	0.623^L^	0.530	0.667^L^
All Slides (153) without 3 readers#	0.705^F^^,^ L	0.627	0.546	0.690^L^

F Performed significantly better (p<0.05) than FluoLED™;

L Performed significantly better (p<0.05) than Lumin™.

# A sensitivity analysis was conducted excluding three readers who had misclassification rates >40%.

Overall, mean examination time was not significantly different across the three LED FMs (114.0 vs. 116.9 vs. 120.5 seconds; Table 4). In sub-group analyses, the Primo Star iLED™ had significantly shorter reading times than the Lumin™ and FluoLED™ with high positive and good quality slides whereas the FluoLED™ had significantly shorter reading times than both Primo Star iLED™ and Lumin™ with poor quality slides. Overall the Olympus BX41TF reference microscope was faster than the LED FMs with a mean reading time of 106.1 seconds across all slide types (data not shown). Mean reading times were longer when excluding three readers with high misclassification rates, and in this group the Primo Star iLED™ (119.4 s) and FluoLED™ (124.8 s) were significantly faster than the Lumin™ (133.2 s; p<0.05).

**Table 4:** Mean slide examination time in seconds among sixteen trainee technicians.

Type of slides (N)	Primostar iLED™	FluoLED™	Lumin™
All slides (160)	114.0	116.9	120.5
Negative (80*)	116.1	118.6	120.1
Low positive (scanty, 1+) (33*)	130.3	131.6	125.4
High positive (2+, 3+) (40*)	97.1^L^	102.1	114.0
Good quality (80)	104.4^F^	124.3	110.0^F^
Poor quality (80)	123.6	109.6^Z^ L	131.2
HIV positives (100)	113.4	120.7	120.1
HIV negatives (60)	115.0	110.7	121.3
All Slides (160) without 3 readers#	119.4^L^	124.8^L^	133.2

F Performed significantly better (p<0.05) than FluoLED™;

L Performed significantly better (p<0.05) than Lumin™.

*7 slides were broken and not read by the experienced reference laboratory technician, and are thus excluded from this analysis.

# A sensitivity analysis was conducted excluding three readers who had misclassification rates >40%.

The overall mean score for the Primo Star iLED™ in the subjective evaluation was significantly higher than the other two LED FMs (4.5 out of 5.0, p<0.05; Table 5). The Primo Star iLED™ also had significantly higher scores for all individual questions except for adaptability of viewing height, in which it performed significantly better than the Lumin™ but not the FluoLED™. Among the LED FMs, the Primo Star iLED™ was ranked by the technicians as being the most preferred for use in daily work at the government health centers, followed by the FluoLED™ then the Lumin™. However, the Olympus BX41TF had the overall highest score and was ranked first for work preference among all four FMs (data not shown).

**Table 5:** Mean scores from the subjective evaluation completed by sixteen trainee technicians.

Question	Primo Star iLED™	FluoLED™	Lumin™
1. How would you rate the adaptability of the viewing height to accommodate your body size and posture?^a^	4.13^L^	3.56^L^	2.81
2. How would you rate the focus mechanism?^a^	4.69^F^^,^ L	3.38^L^	1.88
3. How would you rate the contrast and colour impression?^a^	4.75^F^^,^ L	3.00^L^	2.13
4. How would you rate the homogeneity of fluorescence illumination in the field of view?^a^	4.44^F^^,^ L	3.19^L^	2.00
5. How would you rate the resolution of focus?^a^	4.44^F^^,^ L	3.06^L^	2.06
6. How would you rate the depth of focus?^a^	4.56^F^^,^ L	3.19^L^	2.00
**Overall mean score of the six questions** a	4.50^F^^,^ L	3.23^L^	2.15

a mean score; 1 = very bad, 5 = very good;

F Significantly higher score (p<0.05) than FluoLED™;

L Significantly higher score (p<0.05) than Lumin™.

## Discussion

Across the three LED FMs under evaluation, the Primo Star iLED™ consistently ranked highest and was significantly better than both the FluoLED™ and Lumin™ in misclassification and inter-rater reliability analyzes and in the subjective evaluation. The Primo Star iLED™ also performed at a comparable level to the Olympus BX41TF reference microscope in all analyses except reading times. In general the FluoLED™ performed only marginally worse than the Primo Star iLED™ whilst the Lumin™ compared less favorably and was thought unacceptable for use by technicians. The low specificity results for the LED FMs and the large difference in Olympus BX41TF specificity, between trainee technicians and the experienced technician, demonstrate that a short orientation to FM was insufficient in this setting, This is further emphasized by the low inter-rater reliability and high misclassification rates for all microscopes and indicates that adequate training for LED FMs must be emphasized during country wide roll out.

The high performance of the Olympus BX41TF fluorescent microscope has been well documented [5], [8], [9]. However this microscope may not be affordable or feasible for roll out in low resource settings such as Zambia [11]. As such this evaluation examines three LED FMs to assess which performs best and is most acceptable for use by trainee technicians in district health centers. We show that the Primo Star iLED™ performed similarly to the Olympus BX41TF, except in reading times where the Olympus BX41TF was significantly faster than all LED FMs (data not shown). The Primo Star iLED™ performed comparably even in low quality slides and in slides from HIV-infected patients, which are known to present challenges to readers [6] and are common in a country like Zambia with a high prevalence of HIV and TB/HIV co-infection [7].

In primary analysis the mean slide examination times between LED FMs were not significantly different, however without the three weak readers the Lumin™ was found to have significantly longer reading times than the other two LED FMs. A recent study by Albert at al. comparing the same LED FMs in a reference laboratory in Uganda also found no significant differences in examination time between the Primo Star iLED™ and FluoLED™, and found the Lumin™ to require significantly more time [23]. For all three LED FMs, reading times were faster in this evaluation than for Albert et al.; this could be due to variations in measuring, since the Albert et al study included the time to record smear results as well as to examine the slide. The difference may also result from using technicians from busy government laboratories that have many responsibilities in addition to TB diagnostics, and are thus used to reading slides quickly. Lastly it is worth noting that the fast reading times recorded by technicians in this study could partly explain the high rates of misclassification.

The Lumin™ was found to perform worst in all measures, and was ranked least preferred for use in routine work, reportedly because technicians had difficulty focusing slides. All seven slides broken during the evaluation were on this microscope. These findings are in line with previous literature, where experienced technicians in a reference laboratory found that the FluoLED™ performed significantly better than the Lumin™ and was favored by technicians, because of the quality of the image and ease of focusing [26]. Albert et al, evaluated the same three LED FMs as this study and found no significant difference in diagnostic accuracy between the LED FMs but did report that the Lumin™ was un-acceptable by the technicians because the light intensity was too low, the microscope was not adjustable, had poor contrast and the resolution and depth of focus were unsatisfactory [23].

The low specificity observed here for the three LED FMs highlights that the use of these LED FMs by inexperienced technicians could result in false positive diagnoses. The marked difference in specificity between the experienced technician and the trainee technicians on the Olympus BX41TF suggests that the low specificity observed on the LED FMs is more likely attributable to lack of training than to poor microscope quality. Inter rater reliability across all evaluated microscopes was low compared to other study findings, who report scores on the same microscopes between 0.8–0.9 [14], [20], [24]. These lower numbers could be explained as a function of the readers, who were using the microscopes for the first time after only minimal orientation, whereas the majority of prior studies used experienced readers to perform similar evaluations. As the weighted kappa scores from the Olympus BX41TF were also low this discrepancy was unlikely caused by the LED FMs themselves.

Three technicians had an average misclassification rate much greater (48.1%) than the overall average (23.8%). These readers have 4, 10 and 16 years experience working in a government health center, and read an average of 57 Z-N slides a day on a standard light microscope. These characteristics are not dissimilar to other technicians involved in this evaluation, which indicates that a short orientation to FM microscopes is insufficient for all technicians, even if they possess substantial field experience. Technicians will require tailored training in the use of LED FMs and strong quality assurance and control (QA/QC) programs will need to be implemented as these microscopes are rolled out in government laboratories. Further work should be conducted to assess training requirements in this population. One option for internal QA/QC would be to re-stain and re-examine all FM positive slides with ZN stain as has been recommended in the past. While this may provide diagnostic certainty, it may not be practical in busy government clinics and may create diagnostic challenges when FM and ZN results are discordant. As such, countries that scale up FM will need to develop and implement internal and external QA/QC programs that are feasible in their setting.

The technicians rated the Primo Star iLED™ as the most preferred LED microscope to use after the short orientation; this high user acceptability has been previously been reported in Uganda by Albert et al, [23]. However, it is interesting that the majority (9 out of 16) of these technicians stated in this evaluation that they would prefer to continue using their current light microscope for TB diagnostic purposes. This is likely due to their greater comfort level with light microscopes and a good reminder that there may be some resistance to change as LED FMs are scaled-up; mentorship and further qualitative studies could be implemented to further understand technician acceptability.

Limitations of this study include the brief orientation given to the technicians, which was shorter than has been recommended previously [32]. The idea for a shortened orientation (didactic and hands on practice) was developed following a prior FM evaluation at the same site in Lusaka where two government technicians were trained to use LED FMs. Trainee technicians and an experienced technician read panel tests of slides both pre and post-training. However in the *pre-training* panels both technicians reported AFB concentrations at 100% correlation with the experienced reader in good quality slides and a 95% correlation in slides with varying quality (unpublished data). This data suggested that a brief orientation might be sufficient to enable adequate implementation of LED FMs. Another possible limitation was that one technician had previously used fluorescent microscopes in the past and this may have confounded the impact of the orientation given through this evaluation. However this technician had a comparable misclassification rate (12%) to two other technicians (at 12% and 12.5%) with no previous experience. Lastly, a senior microbiologist (AK) was onsite during this evaluation to lead the orientation and assist technicians if they were unable to focus on the slides, which was only required with the Lumin™. This intervention was added to the protocol after 7 slides were broken, to avoid losing more slides and negatively impacting the evaluation. This could have led to an over-inflation of the Lumin™ performance; however as this microscope ranked lowest in all performance areas this should not impact the overall results of this evaluation.

In conclusion we have demonstrated that the Primo Star iLED™ is the most preferred LED FM, performs better than the FluoLED™ and Lumin™, and is comparable to the Olympus BX41TF when used by laboratory technicians who have received a brief orientation to FMs. The FluoLED™ consistently ranks second in all indicators, which may be of interest as a Primo Star iLED™ microscope currently costs $4825 in high-income countries (compared to about $1750 for countries eligible for reduced pricing [18], [33]), while available literature indicates that the FluoLED™ attachment costs $1977–$3530 depending on model and quantity purchased [18]. We highlight here potential difficulties and resistance that programs may face when introducing new diagnostic tools for tuberculosis at district level. We demonstrate that a short orientation to FM is insufficient for laboratory technicians and recommend that sufficient preparations, proper training with adequate hands-on practice and mentorship are implemented prior to roll out of LED FMs at a national level.
